# Detection of discoloration in diesel fuel based on gas chromatographic fingerprints

**DOI:** 10.1007/s00216-014-8332-4

**Published:** 2014-11-19

**Authors:** Barbara Krakowska, Ivana Stanimirova, Joanna Orzel, Michal Daszykowski, Ireneusz Grabowski, Grzegorz Zaleszczyk, Miroslaw Sznajder

**Affiliations:** 1 Institute of Chemistry, The University of Silesia, 9 Szkolna Street, 40-006 Katowice, Poland; 2 Customs Chamber of Customs Laboratory in Biala Podlaska, 21 Celnikow Polskich Street, 21-500 Biala Podlaska, Poland

**Keywords:** Excise duty components, Partial least squares discriminant analysis, Uninformative variable elimination-partial least squares, Variable selection, Fuel “laundering”, Bootstrapping

## Abstract

In the countries of the European Community, diesel fuel samples are spiked with Solvent Yellow 124 and either Solvent Red 19 or Solvent Red 164. Their presence at a given concentration indicates the specific tax rate and determines the usage of fuel. The removal of these so-called excise duty components, which is known as fuel “laundering”, is an illegal action that causes a substantial loss in a government’s budget. The aim of our study was to prove that genuine diesel fuel samples and their counterfeit variants (obtained from a simulated sorption process) can be differentiated by using their gas chromatographic fingerprints that are registered with a flame ionization detector. To achieve this aim, a discriminant partial least squares analysis, PLS-DA, for the genuine and counterfeit oil fingerprints after a baseline correction and the alignment of peaks was constructed and validated. Uninformative variables elimination (UVE), variable importance in projection (VIP), and selectivity ratio (SR), which were coupled with a bootstrap procedure, were adapted in PLS-DA in order to limit the possibility of model overfitting. Several major chemical components within the regions that are relevant to the discriminant problem were suggested as being the most influential. We also found that the bootstrap variants of UVE-PLS-DA and SR-PLS-DA have excellent predictive abilities for a limited number of gas chromatographic features, 14 and 16, respectively. This conclusion was also supported by the unitary values that were obtained for the area under the receiver operating curve (AUC) independently for the model and test sets.

## Introduction

The steadily increasing level in the consumption of petrol oil worldwide generates considerable profits for the petroleum industry and an increase in the price of petrol oil. Apart from the economic factors, the price of fuel is dependent on the local regulations that define the level of excise tax. In general, many countries apply different levels of excise duty on fuel depending on its usage. For instance, the diesel fuel that is used for heating purposes and in agricultural machinery in Poland has a rebated excise tax that is regulated by law in comparison to the diesel fuel that is used for regular transport. In order to differentiate an oil product with respect to its usage, a dye (red dye Solvent Red 19 or Solvent Red 164) and a marker (Solvent Yellow 124) are deliberately added [1]. The presence of these specific excise duty components neither modifies the physicochemical properties of fuel nor limits its further usage. The substantial financial reward that can be gained from the difference in excise tax has stimulated the illegal practice of removing the excise duty components from rebated fuel and selling it at a higher price. This procedure is known as fuel “laundering” and can be done by an adsorption process using widely available materials. The laundering of commercially available fuel causes a substantial loss in a government’s budget and therefore, the development of analytical procedures for the detection of counterfeit diesel fuel is extremely necessary.

Detection of fuel laundering specifically requires an analytical technique that is capable of revealing chemical changes in the composition of the fuel, since the removal of the excise duty components does not influence its physicochemical parameters. In our previous studies, we developed an analytical methodology to detect any chemical changes before and after a simulated laundering process by using diesel fuel fingerprints that were obtained using fluorescence spectroscopy [2, 3]. It was confirmed that genuine samples can definitely be discriminated from samples after the laundering process. However, only limited information about the chemical composition of complex mixtures can be obtained from their fluorescence fingerprints and that is why gas chromatography coupled with flame ionization detection (GC-FID) was investigated in this study. Gas chromatography (GC) is one of the most popular separation techniques for studying complex petrochemical samples because the chromatograms contain comprehensive chemical information. Gas chromatographic fingerprints [4] are widely used for monitoring quality and/or for identification purposes. These fingerprinting techniques have also been accepted by the World Health Organization (WHO) for the quality assessment of herbal products [5]. To the best of our knowledge, up to the present, GC-FID has not been used for studying the laundering process. The reason is that the excise duty components are not stable and degrade under a high temperature. Their instability was studied and confirmed throughout our experiment when using GC with the nitrogen chemiluminescence detector (sensitive to the presence of compounds containing nitrogen in their structures). Furthermore, the low concentration levels of excise duty components in diesel oil samples make the identification of their peaks among or under the peaks of major sample components difficult. The larger the number of peaks the harder the separation and quantification of analytes with the GC technique is, and this may provide to a failure in the characterization of excise duty components. That is why, the excise duty components are mainly determined with either spectroscopic or HPLC-based techniques. In fact, only the presence or absence of excise duty components is not indicative for a possible laundering process, but the GC-FID fingerprints may contain information about the overall chemical characteristics of samples before and after laundering.

In general, a comparative analysis of chromatographic fingerprints does not require the qualitative or quantitative evaluation of chemical components in samples, but advanced chemometric techniques [6] are required. On the one hand, the costs of the analysis are greatly reduced because no certified reference materials/standards are required and while on the other, important regions in the chromatographic fingerprints that are related to the phenomena being studied (e.g., discrimination/classification of two or more groups of samples) can be found using well-validated multivariate chemometric methods. Once the important regions of chromatographic fingerprints are identified using a variable selection method [7], the corresponding fractions can be collected and further analyzed in detail using an orthogonal chromatographic system or a complementary analytical method. A methodology that combines the fingerprint approach and chemometric analysis has gained popularity in many fields of science and technology in recent years including those such as the development of a method for the estimation of the total antioxidant capacity of green tea [8], the comparative analysis of extraction performance under different conditions [9], the analysis of secondary metabolites in citrus fruits peels [10], classification of petroleum products [11], etc.

In order to investigate whether it is possible to detect diesel fuel laundering, the excise duty components of a number of samples that were obtained from different suppliers in Poland were removed using an adsorption process. Genuine diesel fuel samples and their counterfeit variants were analyzed using gas chromatography coupled with flame ionization detector. Differences between these two groups of samples were studied using the partial least squares discriminant analysis, PLS-DA [12]. The removal of baseline and the alignment of peaks were performed to the sample chromatographic fingerprints using penalized asymmetric least squares approach (PAsLS) [1], and correlation optimized warping (COW) [2], respectively. In order to identify the key regions that are related to the chemical differences of sample groups, the PLS-DA approach was extended with variable selection. In this study, uninformative variable elimination-partial least squares discriminant analysis (UVE-PLS-DA) [13], PLS-DA combined with variable importance in projection (VIP) [14], and selectivity ratio (SR) were investigated [15, 16]. The effect of variable selection was monitored using a bootstrap procedure and the area under the receiver operating curve (AUC) and the sensitivity, specificity, and efficiency for the independent test set were adopted as figures of merit as well.

## Experimental

A total of 31 samples of diesel fuel were collected from different fuel suppliers located in Poland in accordance with the sampling requirements that are specified in the PN-EN ISO 3170:2004 norm. The samples covered the majority of diesel fuel sources that are available for regular customers, and crude oil used for production had a different geographical origin (Poland, Belarus, Lithuania, and The Netherlands). Each investigated sample fulfilled the norm specifications, and thus, could be considered to be representative for a given batch of diesel fuel. Prior to further analysis, the samples were stored at room temperature (ca. 20 °C).

### Registration of the GC-FID fingerprints

The samples of diesel fuel were analyzed twice using a gas chromatographic system (Agilent Technologies 6890N) equipped with a flame ionization detector before and after the removal of the excise duty components. Separation of the components of the mixture was performed using a RTX-5 Restek column, 60 m × 0.25 mm i.d. and 0.25 μm film thickness with helium as carrier gas (1.3 ml min^−1^ constant flow rate, gas purity 5.0). The following temperature program was used: initial temperature 50 °C raised up to 320 °C by 3 °C per minute; total analysis time 100 min. Other settings of applied chromatographic method are as follows: injection mode split (split ratio 20:1); injection temperature 300 °C; injection volume 0.1 μL.

### Processing (laundering) of diesel fuel samples

Every diesel fuel sample was subjected to a specific laboratory treatment that was aimed at removing the excise duty components (the dye and marker). The following laboratory procedure was applied to each genuine diesel fuel sample. Ten milliliters of a sample was placed in a plastic test tube (15 ml) with 2 % of the adsorbent and shaken vigorously for 5 s. Each test tube was shaken two to three times within a 30-min period. Afterwards, each sample was centrifuged at 3500 rpms and the supernatant that was obtained was analyzed as is described in the “Registration of the GC-FID fingerprints” section.

## Theory

### Preprocessing of chromatographic fingerprints

Instrumental signals, e.g., chromatographic fingerprints, consist of three components that are expressed at different levels along the signal’s domain. These are a baseline, a noise, and a pure analytical signal. Each component of the signal has a different frequency range. The noise component has the highest frequency due to the rapid changes within a small amplitude. The baseline component has a very smooth form with a low amplitude of changes and its frequency is the lowest. A pure analytical signal has an intermediate frequency as compared to the frequencies of the baseline and noise components.

Even though the chromatographic conditions are the subject of optimization, chromatographic fingerprints often contain a substantial baseline component. The baseline shape is often irreproducible due to various effects and thus, can influence the construction of multivariate models. It should effectively be removed in order to diminish the negative influence of an overpronounced and fluctuating baseline. To date, many methods have been proposed for removal of the baseline. Among them, the penalized asymmetric least squares method (PAsLS) has found numerous applications. This was the method of choice in our study because of its simplicity and efficiency. More details about the PAsLS method, including definition of its objective function and input parameters, can be found in ref. [17].

In addition to baseline correction, chromatographic fingerprints often require alignment to correct peak shifts. They are the result of different factors that influence the elution time, including the unavoidable effect of column aging. Peak shifts in different chromatograms that originate from the same substance strongly affect the further multivariate data analysis as well as modeling and therefore, their correction is mandatory. The correlation optimized warping approach (COW) is a standard technique that is used for the alignment of peaks [18]. Peak shifts are corrected by stretching and compressing corresponding sections in the target signal and the signal that is being aligned. This is done by maximizing the correlation coefficient between these two signals. In the course of the alignment procedure, the target signal serves as a template for matching chromatographic peaks of every signal from the whole set [19]. An extensive description of the COW method and selection of input parameters is provided in ref. [18].

### Principal component analysis

Principal component analysis (PCA) is a bilinear projection method that is used to visualize and compress multivariate data [20, 21]. With this method, a collection of chromatographic fingerprints, which is organized into a data matrix, is represented as the product of the score and loading vectors, which are called the principal components. The principal components are found by maximizing the description of data variance. A display of score and loading vectors is usually presented for selected pairs of principal components. The proximity of the points on the score plot reflects the chemical similarities among the samples that are described by their chromatographic fingerprints. The loading values (weights) provide information about the relative importance of the variables (fraction(s) of the mixture that is eluted from a chromatographic column within a certain range of elution time) into the construction of a given principal component. Owing to its bilinear character, the score and loading vectors help in assessing any chemical similarities among samples and the loadings indicate the impact of parameters on data structure observed on score projections.

### Partial least squares discriminant analysis

Partial least squares discriminant analysis (PLS-DA) is a variant of the classic partial least squares regression model that aims to discriminate groups of samples [22, 23]. The belongingness of a sample to a certain group is indicated using a categorical dependent variable, *y*. For a two-class discriminant problem, which is within the scope of this study, samples of groups could be coded using a bipolar or a binary dependent vector with elements “−1” and “+1” or “0” and “1” [12].

The PLS-DA model is built using a balanced set of model set samples (the same number of samples from each group) that represent the possible sources of variance characteristic for the two groups of samples well [24]. It is important to emphasize that the selection of the model set samples is crucial for the future prediction of the properties of the PLS-DA model. A uniform scatter of samples over the experimental domain ensures that all sources of variability are taken into account when model is built. The Kennard and Stone or the Duplex algorithm [25] can be used for this uniform selection of the subset for each group of samples separately.

In order to construct the PLS-DA model, its complexity is optimized so that the covariance between a set of latent variables and the response variable, *y*, is a maximum. This is also a key step that has an impact on the future performance of the model. Different cross-validation procedures are frequently used [26] to assess the optimal number of latent PLS-DA variables. Their purpose is to obtain error estimates by perturbing models that are built with an increasing number of latent variables. In general, the cross-validation procedure is an iterative elimination of samples from a model set and an estimation of the prediction error for samples that are removed from the interim model. The final error estimates that are obtained from a series of models with a definite number of latent factors are averaged and displayed as a function of the number of latent factors. Usually, the performance of a discriminant model is presented with figures of merit that are based on the number of correctly recognized samples. Selected figures of merit such as area under the curve, sensitivity, and specificity are defined in the following section. However, the root mean square error of cross-validation is a more sensitive estimate with respect to model complexity and is also an indication of the spread of predicted values.

### Performance of a discriminant model

There are many measures that can characterize the performance of a discriminant model. They are calculated independently for model and test samples. The most popular measure of a model’s performance is the correct discrimination rate, which indicates the number of samples that are correctly recognized using a given discriminant model. Additional figures of merit such as sensitivity (also known as true positive rate, TPR) and specificity (true negative rate, TNR) are derived based on the number of true positive (TP) and true negative samples (TN) as well as false positive (FP) and false negative samples (FN). Sensitivity for a given group of samples is defined as the ratio of the number of true positive samples to the sum of true positive and false negative samples. Specificity expresses the ratio of the number of true negative samples to the sum of the true negative and false positive samples.

The receiver operating characteristic curve, better known as the ROC curve, is an alternative approach to score and illustrate the performance of a discriminant method. The ROC curve summarizes the performance of a discriminant model and displays the trade-off between the true positive rate and false positive rate (1 − specificity) as a function of a model parameter. The convex shape of the ROC curve, i.e., above the line of the unit slope, is an indication of a superior model performance rather than a random guess. The closer the area under the curve (AUC) value is to one, the better the discrimination performance is. Therefore, the perfect discriminant model is characterized by a unitary AUC.

In order to obtain honest estimates of a model’s performance, the bootstrap approach [27] can be adopted. The main idea of the bootstrap approach is to draw, in a random manner, an assumed number of samples from each group in order to form a model set that is used to construct a discriminant model. The remaining samples form the test set and help to estimate the accuracy of the prediction. Since the bootstrap procedure is repeated many times (hundreds of times), the distribution of figures of merit that are being considered is sampled. As a result, the mean value and standard deviation of any parameter that describes the accuracy of the model can be provided in order to illustrate its uncertainty based on multiple random selections of samples. An alternative approach is based on a permutation test. The permutation procedure aims to obtain a reference null distribution (discrimination results are expected to be insignificant) of a certain statistics that are generated from discriminant model that is constructed for the dependent categorical variable that reflects the random assignment of samples to existing groups [29].

### Identification of the relevant explanatory variables for the PLS-DA model

In chemical studies, the number of variables often greatly exceeds the number of samples. That is why chemical data, including chromatographic fingerprints, contain many variables that are noisy and/or unreliable or redundant to the discrimination of the groups. It is known that such explanatory variables affect the prediction properties of the PLS estimator [28] and increase the complexity of a model. Reducing the complexity of a model and obtaining an easier model interpretation and a possible improvement in prediction can be achieved by the variable selection. In the context of PLS-DA, the large number of explanatory variables compared to the number of objects significantly increases the probability of good discrimination by chance. As was illustrated in [29], it is possible to obtain a perfect PLS-DA discrimination for randomly generated data with large ratio of variables to objects. Therefore, the validation of any discriminant model is essential for its practical use. Many variable selection approaches are described in the literature [7]. Three main categories can be described—the filter, wrapper, and embedded methods [28].

#### Uninformative variable elimination-partial least squares

The UVE-PLS wrapper method was proposed to eliminate the uninformative explanatory variables that carry similar information to random variables [13]. In order to distinguish informative variables from uninformative, an experimental data matrix *X* (*m* × *n*) is augmented with a matrix of noisy variables, *N*. The noise matrix, *N*, contains the normally distributed random numbers of a small amplitude (*m* × n*). These noisy variables that have a small variance and negligible covariance with the modeled response variable, in principle, do not influence the construction of the PLS-DA model. During the construction of the PLS-DA model, the stability of the *j*th variable (experimental and artificial), *s*_*j*_, is evaluated based on the jackknifing procedure. The stability of the *j*th variable is defined as the ratio between the mean value and standard deviation of the regression coefficients, *s*_*j*_, is:

1 $$ {s}_j=\frac{{\overline{b}}_j}{\mathrm{std}\left({\mathbf{b}}_j\right)} $$

where, **b**_*j*_ is the vector that contains the regression coefficients of the *j*th variable that was obtained from jackknifing of the PLS-DA model.

Uninformative variables are identified as those with the lower absolute stability of the regression coefficients than the maximal absolute value of the stability of the regression coefficients observed for the noisy variables. Uninformative variables are eliminated from the data and the final model is constructed.

#### Variable importance in projection

Variable importance in projection (VIP) is a simple filter-based variable selection approach that is proposed to assess the relevance of the variables in the PLS-DA model [30, 31]. The importance of the *j*th variable is expressed by its VIP_*j*_ score, which is defined as:

2 $$ {\mathrm{VIP}}_j=\sqrt{\frac{{\displaystyle {\sum}_{f=1}^F{w}_{jf}^2}\cdot {\mathrm{SSY}}_f\cdot J}{{\mathrm{SSY}}_{\mathrm{t}}\cdot F}} $$

where *w*_*jf*_ is the PLS weight value of the *j*th variable and the *f*th component, SSY_*f*_ is the sum of squares of the dependent variable that was obtained from the discriminant model with *f* (*f* = 1, 2, …, *F*) components, *J* is the number of variables, SSY_t_ is the total sum of squares of the dependent variable, and *F* is the number of PLS components evaluated.

The importance of a variable is considered to be highly influential on the PLS model when its VIP score is above 1.0, moderately influential if the VIP score is within the range of 0.8 to 1.0 or if a variable has a small influence—its VIP is below 0.8 [32]. The procedure for the elimination of the variables below a given threshold can be repeated several times in order to reduce the number of variables.

#### Selectivity ratio

The selectivity ratio is another criterion that can be applied in order to filter out irrelevant variables [15, 16]. Irrelevant variables are considered to be those that are not related to the response variable even though they may have large variances. The larger the selectivity ratio of a variable, the greater the correlation with the response variable is. Once the PLS-DA model of a definite complexity is built, the so-called target projection transformation or target rotation is performed so that several PLS-DA components are represented by a target projection score (*m* × 1) vector. Then, a target loading vector (1 × *n*) is obtained from the projection of a model set on to the normalized target score vector. Multiplying the target projection score and loading vectors gives a target projection matrix of dimensions (*m* × *n*). Thus, the original matrix **X** of a model set can be represented as the sum of two matrices—a target projection matrix that contains the information about the PLS-DA model of definite complexity and a residual matrix. A quantitative measure of the selectivity of each variable for the discrimination of groups is the value of the ratio of the sum of squares of the target projection matrix to the residual sum of squares.

#### Bootstrap variants of variable selection methods

A bootstrap strategy was adopted to all of the three methods in order to estimate the effect of the variable selection procedure. The VIP-PLS-DA, SR-PLS-DA, and UVE-PLS-DA models were constructed and validated using an independent test set. The final PLS-DA models for the relevant variables were validated using an independent test set (these samples were not considered in construction of the model) and characterized by the average AUC values for the model set from 1000 bootstrap samples with a replacement and the AUC value for the test set.

For each bootstrap sample of UVE-PLS-DA, a model set containing the chromatographic signals that were selected (with a replacement) from each group was augmented with 100,000 noisy variables that were formed by random numbers drawn from normal distribution (multiplied by a constant factor *c* = 10^−12^). Relevant variables were then selected as those variables with absolute stabilities of their regression coefficients above a cut-off value corresponding to 99.9 % of the maximum value of the absolute stabilities of the regression coefficients for the noisy variables. In fact, 1000 bootstrap samples resulted in 1000 sets of the selected variables and therefore, the variable relevance to the final model is evaluated by the so-called selection frequency, which indicates the percentage of times a variable is selected in the model.

The bootstrap methodology using VIPs and SRs for variable elimination is similar [33]. Basically, it consists of two steps. Firstly, the PLS-DA model with the optimal complexity was constructed for each bootstrap sample and the VIP scores or SRs for the variables were obtained. Secondly, the irrelevant variables were identified as those for which their average values of VIP scores and SRs were below a selected cut-off value. In this study, a cut-off value of 0.8 was found to be optimal for both methods. The threshold value in SR-PLS-DA was selected using the discriminating variables test, DIVA test, and the selectivity ratio plot. In contrast to the SR-PLS-DA method, which was applied only once, the VIP-PLS-DA method was applied three times in a sequential manner in order to reduce the relatively high number of variables that are usually selected when it is only applied once [33].

## Results and discussion

### Preprocessing of the GC-FID fingerprints

**Fig. 1:**
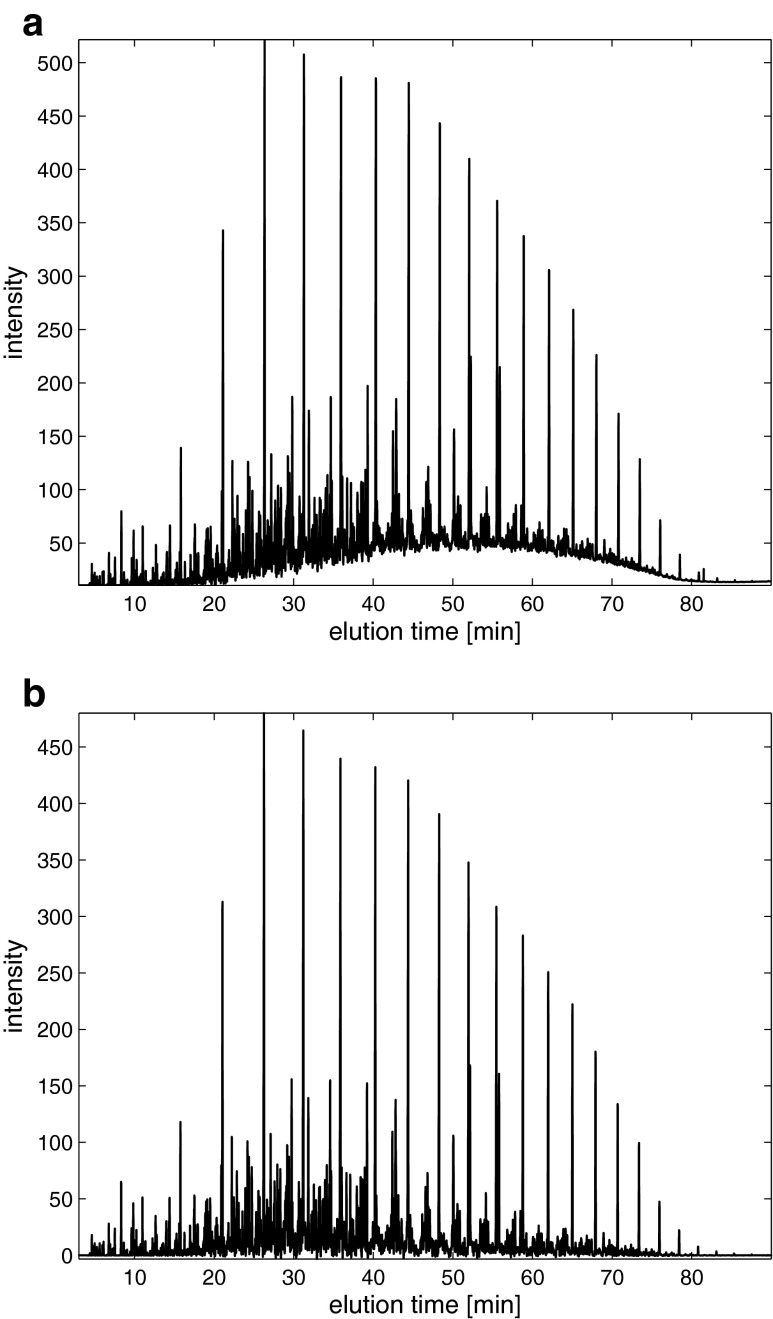
Exemplary GC-FID fingerprint: **a** before and **b** after baseline removal

Because chromatographic signals were collected at a high sampling rate that contained many measuring points, they were resampled using linear interpolation in order to simplify the further modeling. The initial sampling rate of 140,000 sampling points of the GC-FID fingerprints was reduced to 25,000 without a substantial loss of the quality of the signal. Figure 1a presents a typical example of chromatogram obtained from a complex diesel oil sample. Due to limitations of the chromatographic method, many peaks overlap and are not baseline separated. High and sharp chromatographic peaks represent the major components of a sample and are found at characteristic bulge of a baseline. In general, a large number of not fully resolved peaks characteristic for components at relatively low concentrations are found at the base line. Prior to the chemometric analysis, the baseline was removed using the PAsLS method. An acceptable baseline approximation was achieved for *λ* = 10,000 and *p* = 0.001.

A further detailed analysis of the GC-FID fingerprints collected also revealed a problem with the peak shifts. The correlation optimized warping method was used in order to correct the peak shifts. In this study, the reference chromatogram was selected as described in [19]. A different number of sections (starting with the length of a section corresponding to an average peak width of 50 sampling points) and values of the slack parameter were evaluated. A satisfactory alignment was achieved when the alignment was carried out for 250 sections (100 sampling points per section) and the slack parameter was equal to three for the majority of the GC-FID fingerprints. The signals after a baseline correction and alignment are presented in Fig. 1b.

**Fig. 2:**
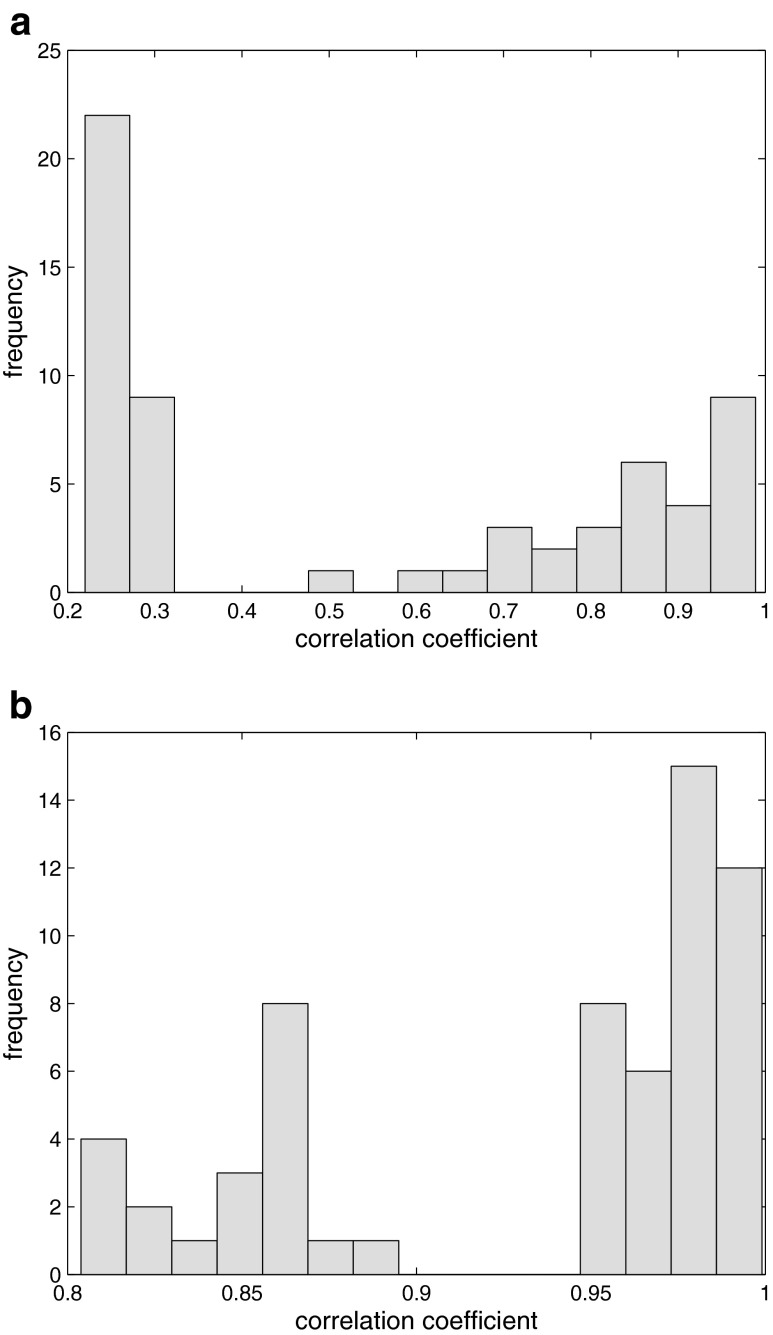
Histograms of correlation coefficients calculated between each chromatographic fingerprint and a target signal: **a** before and **b** after alignment using COW

The smallest value of the initial correlation coefficient between a signal and target was about 0.220, whereas the largest value was 0.989. A few fingerprints, which had relatively low correlation coefficients with respect to the target signal (compared to majority of signals), were aligned again using different input parameters. In general, most of the GC-FID fingerprints were characterized by correlation coefficients that were higher than 0.8 after the alignment procedure. The smallest correlation coefficient that was observed was 0.804 and the largest value was 0.999. To illustrate the effect of the alignment procedure, histograms of the initial and the final (after alignment) correlation coefficients that were computed between each fingerprint and the target signal are presented in Fig. 2.

Preprocessed GC-FID fingerprints of genuine and counterfeit samples were further modeled using multivariate discriminant methods in order to verify the possibility of their discrimination.

### Exploration of the GC-FID fingerprints

**Fig. 3:**
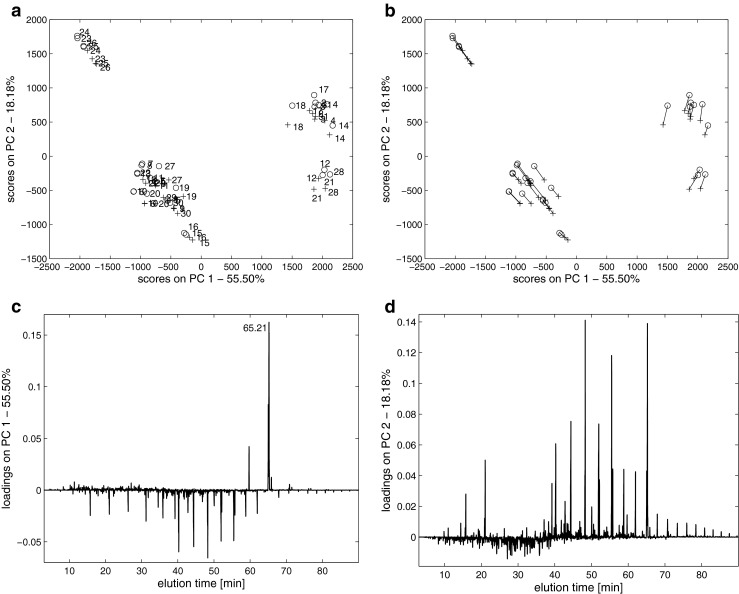
Projection of samples (scores) onto space defined by first two principal components: **a** samples denoted as *plus sign* are authentic and samples denoted as *empty circle* are after the laundering process and **b** illustration of corresponding pairs of samples authentic *plus sign* and counterfeit variant *empty circle* (i.e. after the laundering process). Loadings as a function of retention time for: **c** PC 1 and **d** PC 2

Potential differences in the chemical composition of diesel fuel samples were studied using the PCA method. Preprocessed GC-FID fingerprints (baseline corrected, aligned, and mean-centered) of genuine and counterfeit samples can be modeled with two principal components that describe 73.68 % of the total data variance. Projection of the samples onto the space that was defined by the first two principal components allows some conclusions about their chemical similarities to be drawn. Each point on the PC 1-PC 2 projection (Fig. 3a) represents one GC-FID fingerprint (sample). Genuine and discolored samples were denoted as “+” and “○”, respectively. In Fig. 3b, for a better clarity of presentation pairs of samples authentic and counterfeit are connected with a line. Two groups of diesel fuel samples can be observed along the PC 1 axis and another two groups along the PC 2 axis. By analyzing the score projections in Fig. 3a, one can conclude that the laundering process itself is not substantially influential for the separation of the samples along PC 1 and PC 2. The corresponding loading plots in Fig. 3c, d, which show the ranges of the elution times, indicates the two chromatographic peaks that are responsible for the differences between the two groups of samples. These two peaks correspond to the mixtures that were eluted at ca. 65.21 and 65.24 min. They can be attributed to the methyl esters of fatty acids (FAME). The FAME compounds cannot be considered as possible markers for the laundering process. They are deliberately added to diesel oil during its production and are present in the studied samples regardless the laundering process. In Poland, any manufacturer is allowed to add up to 7 % (*V*/*V*) of FAME, but the differences in the total amount of FAME from batch to batch of diesel oil depend on the temporary production and economic requirements. The group of diesel oil samples characterized by positive score values along PC 1 (see Fig. 3a) contains FAME, the content of which varies in the range of 4.1 % (*V*/*V*) and 5.3 (*V*/*V*). Other variability sources such as different producers, origin of crude oil used in the production process, production process itself have an impact on forming clusters of samples.

Unfortunately, no groups of samples that underwent the laundering process were revealed in the other score projections built for consecutive pairs of the selected principal components. Therefore, the discrimination of groups along with the directions that describe the largest data variance in the experimental space is impossible. However, this does not necessarily mean that the supervised discrimination between the groups is also impossible. For this reason, the next step of the chemometric processing of GC-FID fingerprints was aimed at the construction of a supervised PLS-DA model to possibly support the differences in the chemical composition of genuine and counterfeit diesel fuel samples. On the other hand, Fig. 3a provides evidence that chemical composition of laundered samples is different from chemical composition of authentic ones. In Fig. 3a, corresponding pairs of samples (authentic samples marked as “+” and counterfeit samples marked as “○”) are connected with a line. Samples after laundering are shifted with respect to its authentic variant. Since counterfeit samples are found in the same clusters, chemical composition is modified in a moderate degree and most probably concern components at low concentrations (minor components).

### Construction of the PLS-DA model

Prior to the construction of the PLS-DA model, model diesel fuel samples were selected according to the following scheme. A total of 21 samples from the genuine group of diesel fuel samples were chosen using the Kennard and Stone algorithm in order to cover all of the possible sources of variability [25]. Genuine diesel fuel samples were coded as “+1” to reflect the presence of the excise duty components. The second group of the counterfeit diesel fuel samples contained the same samples, but after the laundering process. The samples of this group were coded as “−1” in order to indicate the absence of the excise duty components. The remaining ten diesel fuel samples and their ten counterfeit variants formed the test set and were used to characterize the predictive abilities of the model. The optimal number of latent factors, which were required to build a PLS-DA model for each bootstrap sample (selected with replacement from the model set), was selected based on the leave-one-out cross-validation procedure. As is indicated in Table 1, the PLS-DA model helps in discriminating all of the samples from the test set correctly. The excellent discrimination results that were obtained from the PLS-DA model support the hypothesis that the process of diesel fuel laundering can be detected based on the diesel fuel GC-FID fingerprints.

**Table 1:** Performance of partial least squares discriminant models with and without the variable selection scheme

Type of model	No. of variables	*f*	AUC model set	AUC test set	Sensitivity, specificity model set (%)	Sensitivity, specificity test set (%)
PLS-DA	25,000	6	1.000	1.000	100.00 100.00	100.00 100.00
UVE-PLS-DA	14	9	1.000	1.000	90.00 100.00	100.00 100.00
VIP-PLS-DA	265	6	0.996	0.970	95.24 95.24	90.00 90.00
SR-PLS-DA	16	3	1.000	1.000	100.00 100.00	100.00 100.00

**Fig. 4:**
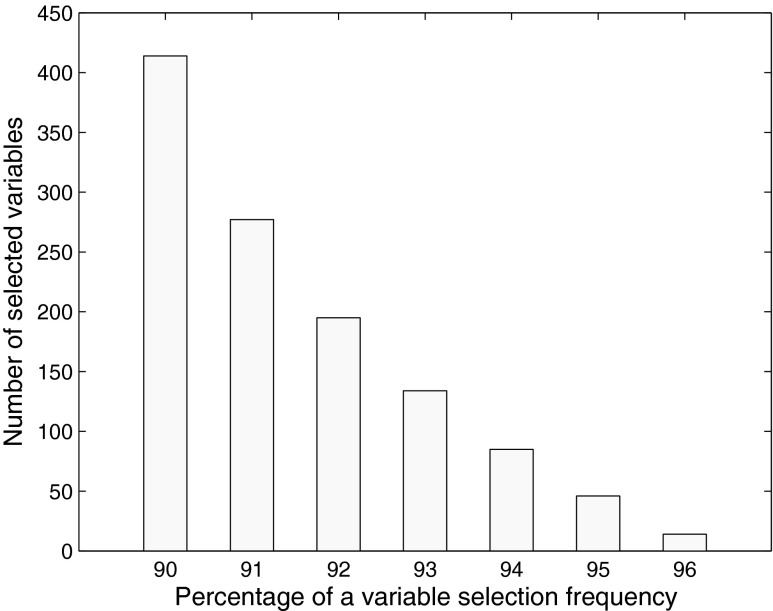
The number of relevant variables identified using the UVE-PLS-DA approach as a function of percentage of the variable selection frequency

The bootstrap variable selection methods were then considered in order to avoid the possibility of presenting results of an overfitted discriminant model. Figure 4 illustrates the number of relevant variables that were selected from UVE-PLS-DA as a function of the percentage of the variable selection frequency.

In 96 % of all of the bootstrap subsets (1000 subsets drawn with replacement), only 14 variables out of 24,966 were found to be the most relevant. They corresponded to the mixtures that were eluted from the chromatographic column after 23.937, 23.940, 23.944, 24.786, 24.789, 24.793, 24.796, 25.398, 25.402, 27.556, 27.559, 27.563, 40.881, and 40.884 min. A list of potential chemical components, eluted from a column at selected retention times, is provided in Table 2. Identification of the chromatographic peaks described in Tables 2 and 3 was based on retention time index obtained from GC-MS (GC Agilent Technologies 7890A with MS 5975C detector) and supported by the NIST 2011 library. Compared to the corresponding genuine fuel samples, these components were found at lower levels in the laundered fuel samples.

**Table 2:** Identification of chemical compounds found in mixtures eluted at retention times indicated as relevant using the UVE-PLS-DA approach

Peak number	Retention time [min]	Possible compound
1	23.937 23.940 23.944	Benzene, 1-methyl-3-propyl formula: C_10_H_14_
2	24.786 24.789 24.793 24.796	Benzene, 1-methyl-4-propyl formula: C_10_H_14_
3	25.398 25.402	Benzene, 1-ethyl-2,4-dimethyl formula: C_10_H_14_
4	27.556 27.559 27.563	Benzene, 1,2,3,5-tetramethyl formula: C_10_H_14_
5	40.881 40.884	*n*-Paraffin C_14_

**Table 3:** Identification of chemical compounds found in mixtures eluted at retention times indicated as relevant using the SR-PLS-DA approach (NI—not identified)

Peak number	Retention time [min]	Possible compound
1	7.052 7.055	NI
2	23.982 23.985 23.989 23.991 23.996 23.999	4-Ethyloheptan formula: C_9_H_20_ or 1-octanol, 2-butyl formula: C_12_H_26_
3	32.292 32.296 32.299	Phytol formula: C_20_H_40_O
4	32.891 32.894 32.898	Compounds containing oxygen, e.g., 1-propene, 2-nitro-3-(1-cyclooctenyl) formula: C_11_H_17_NO_2_
5	38.508	NI
6	47.058	Pentadecane, 3-methyl formula: C_16_H_34_

The final UVE-PLS-DA model was built and validated with an independent test set using the selected variables. The bootstrap procedure with a replacement was again carried out in order to evaluate the effect of the variable selection. The UVE-PLS-DA model was characterized by a unitary average AUC value for the model and test set. Only one sample from the model set was recognized incorrectly as a genuine sample. This results in a sensitivity of a 90 % and a highest specificity of 100 %. The sensitivity, specificity, accuracy, and correct classification rate for the final discriminant model with nine PLS factors and for the test set samples are presented in Table 1.

The final discriminant model with six latent factors that was built for 265 variables that were selected with the bootstrap VIP-PLS-DA procedure presented average AUC values of 0.996 and 0.970 for the model and test set, respectively. One sample in each group of the model and test set was incorrectly recognized using the final PLS-DA model. Both figures of merit (sensitivity and specificity) for the model set were equal to 95.24 %, whereas they were at a level of 90.00 % for the test set samples.

As was mentioned earlier, the average selectivity ratio for each variable was obtained from the bootstrap procedure. Irrelevant variables were identified as those with an absolute average selectivity ratio below the threshold value of 0.8. Only 16 variables were recognized as relevant. They correspond to the mixtures that were eluted at ca. 7.052, 7.055, 23.982, 23.985, 23.989, 23.991, 23.996, 23.999, 32.292, 32.296, 32.299, 32.891, 32.894, 32.898, 38.508, and 47.058 min (see Table 3).

The optimal PLS-DA model that was constructed for 16 variables had only three latent factors and offered an excellent discrimination performance for the model and test set samples. Once again, the unitary average AUC values for the model and test set as well as sensitivities and specificities of 100 % were obtained.

### A comparison of the results that were obtained from variable selection approaches

In general, it is not easy to decide which variable selection method will show the best performance for a given discriminant problem. A number of variable selection methods for PLS-DA have been developed for this purpose. Here, we considered two filter methods and one wrapper method. UVE-PLS-DA and SR-PLS-DA are methods that are specially designed to select variables that correlate with the response variable, even though they can have low variances. Both methods achieve this through the different mechanisms that were described earlier. In this study, they presented the best predictive abilities (sensitivity and specificity of 100 % for the test set) and a small number of selected variables in comparison with the VIP-PLS-DA. In our study, even though a sequential scheme of variable selection was adopted in VIP-PLS-DA, a large number of variables were found to be important. These variables are in fact the olefin substances that are present in the highest concentrations in diesel fuel and definitely present large absolute sizes (have large variances in the PLS-DA model), but from a chemical point of view, they are not necessarily related to the laundering process. Moreover, the VIP-PLS-DA method showed the worst predictive performance among the three variable selection discriminant methods. The other two methods, UVE-PLS-DA and SR-PLS-DA, found two sets of important variables. A closer look of these sets of variables indicates that the variables that were selected by SR-PLS-DA have a clear chemical interpretation since these are polar substances, the concentrations of which decrease during the adsorption laundering process.

## Conclusions

We came to several important conclusions in this work. The data exploration that was performed using PCA did not show any characteristic distribution of chromatographic fingerprints of diesel fuel samples with respect to the differences in the sample contents. On the other hand, all three variable selection methods, UVE-PLS-DA, SR-PLS-DA, and VIP-PLS-DA, show potential in the detection of laundering process. Among them, VIP-PLS-DA presented the worst predictive performance and the largest number of selected variables. The other two methods showed a sensitivity, specificity, and efficiency of 100 % for the test set using a small number of variables (14 and 16). A closer look at the variables that were selected by both methods indicated that the variables that were obtained from SR-PLS-DA have a straightforward chemical interpretation. These are polar substances, the concentration of which decreases during the adsorption laundering process. Therefore, it appears that PLS-DA with the variables that were selected using their selectivity ratios is the method of choice for the detection of illegal diesel fuel discoloration. Although a larger number of commercially available diesel fuel samples should be considered in order to definitely determine the general use of the methodology, these results indicate the potential and practical use of proposed method.
